# Feasibility and acceptability of home-based neonatal hyperbilirubinemia screening by community health workers using transcutaneous bilimeters in Bangladesh

**DOI:** 10.1186/s12887-023-03969-4

**Published:** 2023-04-03

**Authors:** Mahbubur Rahman, Farjana Jahan, Sk Masum Billah, Farzana Yeasmin, Musarrat Jabeen Rahman, Tania Jahir, Sarker Masud Parvez, Jyoti Bhushan Das, Ruhul Amin, Khobair Hossain, Hannah Grant, Rezaul Hasan, Gary L. Darmstadt, Md. Mahbubul Hoque, Mohammod Shahidullah, Muhammad Shariful Islam, Sabina Ashrafee, Eric M. Foote

**Affiliations:** 1 Environmental Interventions Unit, Infectious Diseases Division, International Centre for Diarrhoeal Disease Research, Bangladesh (icddr, b), 68 Shaheed Tajuddin Ahmed Sarani, Mohakhali, Dhaka, 1212 Bangladesh; 2 Maternal and Child Health Division, International Centre for Diarrhoeal Diseases Research, Bangladesh (icddr, b), 68 Shaheed Tajuddin Ahmed Sarani, Mohakhali, Dhaka, 1212 Bangladesh; 3 Faculty of Medicine and Health, School of Public Health, University of Sydney, Sydney, Australia; 4 John Hopkins Bloomberg School of Public Health, Baltimore, MD 21205 USA; 5 College of Medicine, Nursing & Health Sciences, University of Galway, Galway, Ireland; 6 Children’s Health and Environment Program, Child Health Research Centre, The University of Queensland, South Brisbane, Brisbane, QLD Australia; 7 Prematurity Research Center, Department of Pediatrics, Stanford University School of Medicine, Stanford, CA USA; 8 Department of Neonatology, Bangladesh Shishu Hospital & Institute, Dhaka, Bangladesh; 9 Bangabandhu Sheikh Mujib Medical University, Dhaka, Bangladesh; 10 National Newborn Health Program (NNHP) and Integrated Management of Childhood Illness (IMCI), Directorate General of Health Services, Dhaka, Bangladesh

**Keywords:** Community Health Worker, Transcutaneous Bilirubin, Neonatal Hyperbilirubinemia, Phototherapy, Feasibility, Acceptability

## Abstract

**Background:**

Universal screening for neonatal hyperbilirubinemia risk assessment is recommended by the American Academy of Pediatrics to reduce related morbidity. In Bangladesh and in many low- and middle-income countries, there is no screening for neonatal hyperbilirubinemia. Furthermore, neonatal hyperbilirubinemia may not be recognized as a medically significant condition by caregivers and community members. We aimed to evaluate the acceptability and operational feasibility of community health worker (CHW)-led, home-based, non-invasive neonatal hyperbilirubinemia screening using a transcutaneous bilimeter in Shakhipur, a rural subdistrict in Bangladesh.

**Methods:**

We employed a two-step process. In the formative phase, we conducted eight focus group discussions with parents and grandparents of infants and eight key informant interviews with public and private healthcare providers and managers to explore their current knowledge, perceptions, practices, and challenges regarding identification and management of neonatal hyperbilirubinemia. Next, we piloted a prenatal sensitization intervention and home-based screening by CHWs using transcutaneous bilimeters and evaluated the acceptability and operational feasibility of this approach through focus group discussions and key informant interviews with parents, grandparents and CHWs.

**Results:**

Formative findings identified misconceptions regarding neonatal hyperbilirubinemia causes and health risks among caregivers in rural Bangladesh. CHWs were comfortable with adoption, maintenance and use of the device in routine home visits. Transcutaneous bilimeter-based screening was also widely accepted by caregivers and family members due to its noninvasive technique and immediate display of findings at home. Prenatal sensitization of caregivers and family members helped to create a supportive environment in the family and empowered mothers as primary caregivers.

**Conclusion:**

Adopting household neonatal hyperbilirubinemia screening in the postnatal period by CHWs using a transcutaneous bilimeter is an acceptable approach by both CHWs and families and may increase rates of screening to prevent morbidity and mortality.

## Background

Neonatal hyperbilirubinemia is one of the most common neonatal conditions demanding medical attention in the first week after birth. [1–5] Worldwide, an estimated 14.1 million newborns (10.5% of live births) require phototherapy for neonatal hyerpbilirbinemia; of these, 6 million do not have access to treatment and 2.4 million of those without access to treatment are in Central and South Asia. [6, 7] In low-resource settings, where the majority of births occur outside facilities and access to monitoring and laboratory testing is limited, many proven diagnostic and treatment strategies are not available. [8] In Bangladesh, and in many low- and middle-income countries, there is no universal screening approach or pre-discharge assessment of neonatal hyperbilirubinemia risk. Approximately half of mothers deliver at home, and only 7% of home births are seen by a medically trained provider within 48 h of birth. Hospital stay for those who deliver at a hospital by normal vaginal delivery is 4–8 h. Thus, hospital-based screening for neonatal hyperbilirubinemia is not currently feasible,[9] emphasizing the need for household-level screening.

Timely screening and management of neonatal hyperbilirubinemia are critical to prevent adverse neurologic consequences. [10–13] To increase the early identification of neonatal hyperbilirubinemia, point-of-care screening that is easily administrable by community health workers (CHWs) at the household may increase access and improve screening rates. [14, 15] However, there is currently no evidence of the acceptability and feasibility of household-based screening of neonatal hyperbilirubinemia in low- and middle-income country (LMIC) settings, and information is lacking on how such interventions can best be designed for acceptability in LMICs. Moreover, information on caregivers’ current awareness and practices of screening and management of neonatal hyperbilirubinemia are important for designing and implementating an effective community-based screening method. [16, 17].

We report the acceptability and operational feasibility of a CHW-led, home-based, sensitization and neonatal hyperbilirubinemia screening using a transcutaneous bilimeter in rural Bangladesh. We present findings on: (1) Existing knowledge, perceptions and management of neonatal hyperbilirubinemia in rural Bangladesh, (2) Prenatal sensitization on neonatal hyperbilirubinemia, including CHW and beneficiary perspectives on feasibility and acceptability, and (3) Operational feasibility of home-based neonatal hyperbilirubinemia screening using a transcutaneous bilimeter.

## Methods

### Study approach

This was a qualitative research study conducted at the formative and pilot phase of a cluster randomized controlled trial to evaluate the effectiveness of CHW-led, home-based, neonatal hyperbilirubinemia management in Sakhipur sub-district of Tangail district, Bangladesh. The study was conducted in two steps: (1) Formative exploration of neonatal hyperbilirubinemia screening and management to design the intervention, and (2) Assessment of acceptability and operational feasibility of community-based screening using a transcutaneous bilimeter.

#### Formative research

The first step in designing community-based neonatal hyperbilirubinemia management was to identify intervention parameters. Once the intervention parameters were identified, a list of desired practices and a set of research questions (Table 1) that would help in designing the intervention component and mode of delivery were developed through literature review and discussions with experts. We conducted qualitative exploratory research to answer the research questions. The field team conducted eight focus group discussions with community participants (parents and grandparents of infants < 12 months old) and eight in-depth interviews with health care providers (HCP) and managers (Table 2). We selected grandparents, along with parents, as they are important decision-makers and engaged with child rearing in the family. We selected parents of infants < 12 months old for easier recall of their most recent experiences with child rearing. We included both male and female participants because females are the primary caregivers, and males are important decision-makers for financial or medical issues such as child health care-seeking. We identified and listed 40 eligible parents and grandparents by visiting households and inviting them to participate in a focus group discussion.

The health sector participants were selected based on their engagement in providing services to manage and treat neonates. We included participants from each tier of the health service sector of a district, and sub-district union and village levels and from both public and private sectors. Participants from the private sector included pediatricians and informal HCPs including village doctors (*gram daktars)* and providers practicing homeopathic medicine. The typical *gram daktar* is an unlicensed, self-appointed, health service provider, usually with some background experience working as an allopathic medicine pharmacist or as an assistant to formally qualified medical professionals. We also interviewed local traditional healers practicing Ayurveda treatment (Table 2).

**Table 1:** Research questions to be answered by the formative research

1. **Community outreach**	
a. What are the current community perceptions and practices for neonatal jaundice? b. What are the perceived barriers and challenges faced by direct community-level health service providers in reaching out to the community c. What outreach activities should be implemented? d. How should local healthcare infrastructure be supported to adopt the program? e. How can the newly trained CHWs be integrated into the care infrastructure?	
2. **Breastfeeding and neonatal jaundice module development**	
a. How should community-level implementation of breastfeeding and the neonatal jaundice awareness package be adapted to the conditions in sub-district? b. How should the information for mothers and families best be presented to maximize understanding?	
3. **Developing intervention delivery and evaluation modality**	
a. How should content for tablets used by CHWs during home visits be designed? The tablets will function both as job aids, and as monitoring tools. CHWs will use the tablets to (1) guide decision making; (2) facilitate communication of complex messages including showing images on the tablet to mothers; (3) collect information on the outcome of the home visit, uptake of interventions b. How should CHW performance be monitored and evaluated?	

**Table 2:** Data collection tools and sampling method

Study phase	Tools applied	Sample	Number	Participants	Total participants
Formative research	Community Participants				
	Focus groups	Grandparents	4	4 villages from 2 unions and 1 from each village	29
		Parents	4	4 villages from 2 unions and 1 from each village	34
	Health Sector Participants				
	In-depth-interviews	Health care service providers		Physicians	3
				Nurse	1
				Community Health Care Providers (CHCP),	1
				Sub Assistant Community Medical Officer (SACMO	1
				Informal: *Gram daktar* (village doctor)	1
				Informal: homeopathy doctor	1
Endline Qualitative research	In depth interview	Study implementers Caregivers	10 14	Community health worker Mothers	10 14
	Focus group discussion	Study implementers Caregivers	1 4	Community health workers Parents and grandparents	8 30

#### Intervention design

The formative research findings were presented at a planning workshop at the National Newborn Health Program and Integrated Management of Childhood Illness, Directorate General of Health Services in July 2019. The purpose was to encourage community engagement and ensure that the intervention was aligned with the government health system. We invited representatives from the Ministry of Health and Family Welfare, academics, neonatologists, obstetricians, and development partners working in maternal and child health. The planning workshop resulted in the development of an intervention plan and a conceptual framework for the intervention impact pathway.

We developed a training manual and educational session module to deliver the prenatal sensitization messages. During this development, two additional workshops were conducted to design the content and to determine how best to illustrate the desired messages through pictures.

The creative briefs were discussed with local artists and printers who produced drafts of the materials. The draft manual and educational modules were presented at a workshop and revised based on the workshop recommendations. They were then validated by experts and approved by the National Newborn Health Program.

#### Community Health Worker-led prenatal sensitization

##### Selection of CHWs

CHWs were the focus of this intervention package. They are an existing cadre in the health system under Community-based Health Care. Men and women are recruited from the local community to become CHWs and are trained to conduct home visits. The selection of CHWs for this study was based on the existing cadre criteria. However, we selected only females because the intervention was designed for mothers and neonates, and female CHWs would be more acceptable to pregnant women and newly delivered mothers at the household level.

##### Prenatal session module and approach

**Fig. 1:**
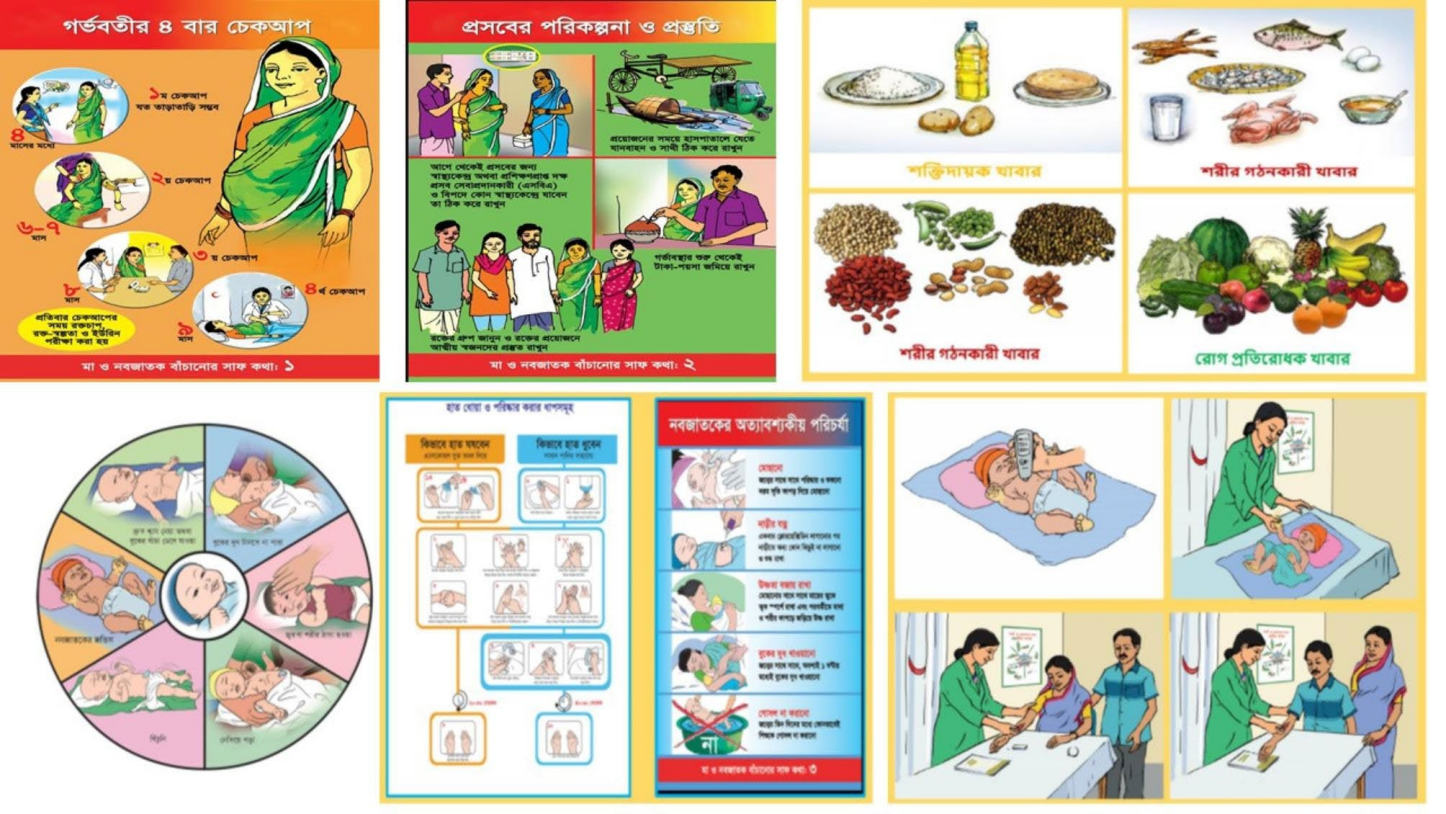
Prenatal educational session content for mothers (icddr,b, National Newborn Health Program, Directorate General of Health Services, Bangladesh)

The International Centre for Diarrheal Disease Research, Bangladesh (icddr,b) team in collaboration with the Directorate General of Health Services, academics, and clinicians from Dhaka Shishu Hospital and Bangabandhu Sheikh Mujib Medical University developed the contents of the prenatal sessions. To deliver the prenatal sessions, we developed a pictorial flip chart (Fig. 1) that was designed to be understandable for the mothers. During home visits, CHWs counseled mothers and families on: (1) the use of preventive care, such as routine antenatal check-ups at health clinics, (2) birth preparation, including planning for a clean and safe delivery, (3) newborn care, such as early and exclusive breastfeeding, hypothermia prevention, and umbilical cord care, (4) recognition of maternal and newborn danger signs and timely care-seeking, and (5) signs and symptoms of neonatal hyperbilirubinemia and where to go if treatment is needed. CHWs also encouraged family members to inform them when women are in labor.

#### Training of CHWs

We designed a five-day training for the CHWs for inclusion in the government training program. The contents of the training and mode of delivery were designed to allow active participation of the CHWs while not overburdening them. The training was conducted in two phases: theoretical and practical, including hands-on training, video messages, and role play. The first three days of training described the maternal and child health conditions in Bangladesh. This included the responsibilities of CHWs, pregnancy care and maternal danger signs, delivery planning and safe delivery preparations, essential newborn care, breastfeeding and management of breastfeeding problems, postpartum services and the responsibilities of CHWs, neonatal jaundice types and symptoms, neonatal jaundice risk and management, descriptions of equipment and materials associated with jaundice management, and the sick newborn referral system and its components. In the second phase, CHWs received training on general examination of the newborn infant, physical examination for neonatal jaundice and assessment of neonates and mothers for other danger signs, [18] neonatal hyperbilirubinemia signs and symptoms, screening with transcutaneous bilimeter and management using mHealth application, assessment of breastfeeding, and management of breastfeeding problems.

#### Home-based prenatal sensitization

The CHWs conducted a visit at least monthly with the enrolled pregnant mothers. In total, 278 mothers were enrolled between September 2019 to March 2021 and received prenatal sessions by CHWs. Due to nationwide lockdown for the Covid-19 pandemic, we halted our study activities from March 26, 2020 - September 30, 2020. After assessing feasibility, we resumed our field activities from November 1, 2020 following all icddr,b guidelines for resumption of field activities during the Covid-19 pandemic. Each CHW was assigned to one cluster which comprised between 26 and 32 households. During the prenatal period, CHWs counseled mothers on pregnancy care and maternal danger signs, delivery planning and safe delivery preparations, essential newborn care, breastfeeding, and signs, symptoms and management of neonatal hyperbilirubinemia. As a part of our CHW-led perinatal education sessions, they emphasized early initiation and exclusive breastfeeding up to six months and maternal awareness of identification and care-seeking for neonatal hyperbilirubinemia.

#### Neonatal hyperbilirubinemia screening by transcutaneous bilimeter

**Fig. 2:**
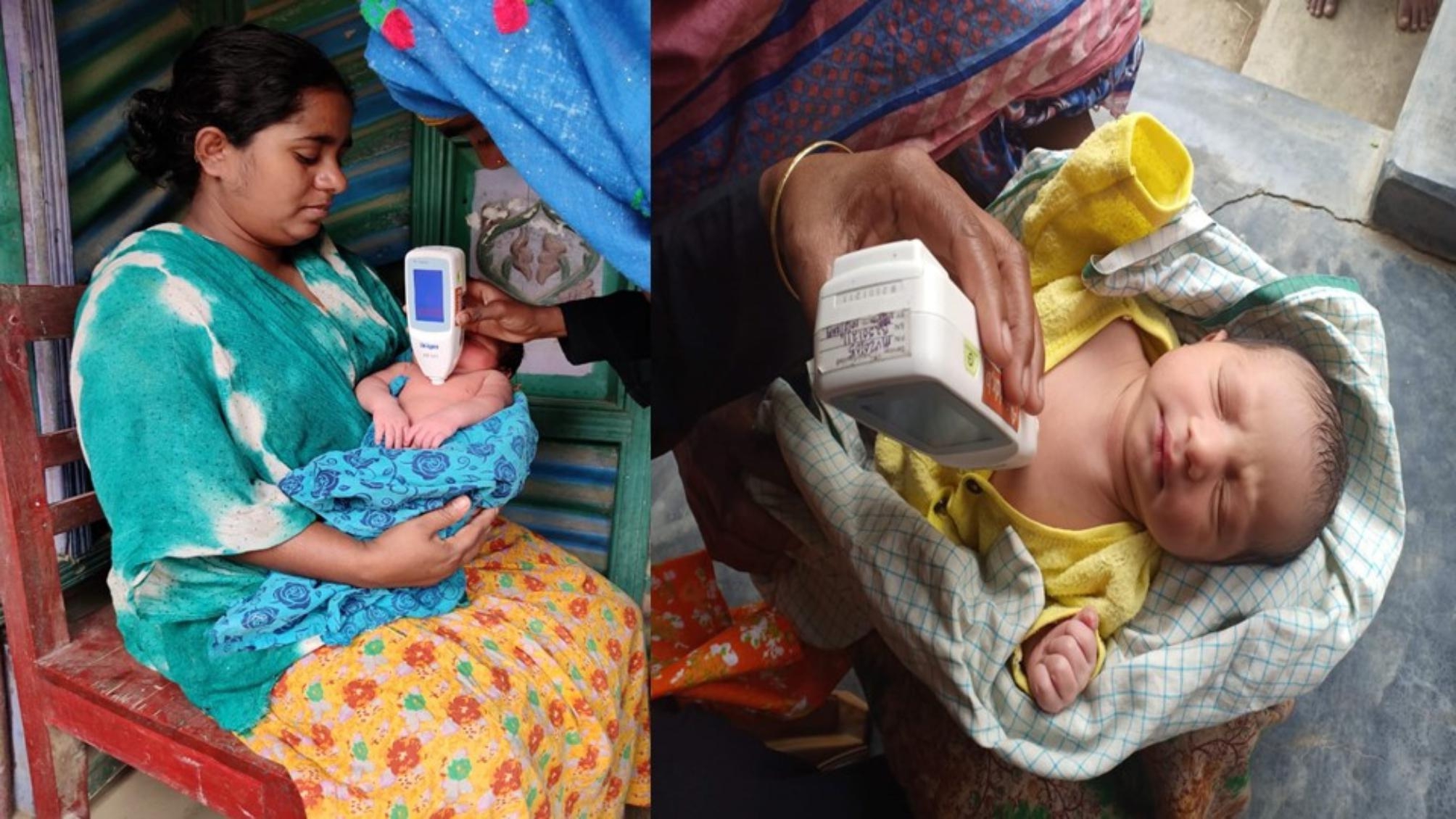
Home based neonatal jaundice screening by community health workers with transcutaneous bilimeter. (source: icddr,b)

CHWs conducted scheduled home visits within 48 h of vaginal births and within 24 h of discharge of newborns born via caeasarean section and then daily for 3 consecutive days to measure the bilirubin with a transcutaneous bilimeter. Total bilirubin was measured using the Draeger JM-105, a non-invasive handheld device that measures the transcutaneous bilirubin (TcB) level of a newborn painlessly by pressing it over the sternum (Fig. 2). [19] The CHWs took three consecutive readings for each screening and input the average TcB in the CommCare application. [20] CommCare is a mobile-based application designed to support frontline workers to deliver care outside of facilities. It has been used in survellinace, community-based antenatal and postnatal care, and management of malnutrition in different settings. [21–24]

#### Assessment of acceptability and operational feasibility

We applied purposive and convenience sampling techniques to select the participants for qualitative assessments. We conducted 14 in-depth interviews with mothers, four focus group discussions with parents and grandparents, 10 in-depth interviews with CHWs and four key informant interviews with field supervisors to evaluate operational feasibility including the barriers and facilitators of community-based neonatal hyperbilirubinemia screening using a transcutaneous bilimeter.

### Data collection

The field team included members with anthropology and social sciences backgrounds who had extensive experience conducting qualitative interviews. All data were recorded on digital audio recorders. The team took detailed notes during the group discussions. All in-depth interviews were conducted one-to-one, and on average each interview duration was 40–45 min. In each focus group discussion, three researchers were involved— one facilitated the discussion, one took notes and the third one managed the logistics by reminding participants about the discussion time, seating arrangement, controlling crowds, and distributing refreshments to the participants. Focus group discussions were 50–60 min in duration on average. All questions were asked in the native Bangla language.

### Data analysis

To analyze the data from the formative research and qualitative study, the research team performed deductive and inductive thematic content analysis following grounded theory [25]. An inductive process was predominantly used in the formative phase to generate themes through close reading of all transcripts. A deductive process was predominantly used in the later phase of qualitative assessment when the intervention was implemented and we sought to explore the acceptability and feasibility of the intervention. Researchers recorded in-depth interviews and focus group discussions using digital audio recorders and took notes in hardcopies for any important findings. Later, we transcribed the audio recordings verbatim into Bengali in the word processor and coded the text using the qualitative data analysis software Atlas.ti (version 5.2). For coding in Atlas.ti, we prepared an initial set of themes after reading all the transcripts, summaries and written notes. Five team members coded each transcript based primarily on generated themes. During the coding process, the team also identified and included other codes. Once all the codes were identified, we sorted and collated coded data extracts into generated themes. After sorting all the coded data into the various themes, we revisited the themes and discarded those not supported sufficiently by the data.

### Ethical consideration

This study was conducted under the ethical principles of the Declaration of Helsinki. Written informed consent was obtained from the participants prior to the interview and recording. Participants were offered the opportunity to read the consent form fully and ask and receive answers to any questions before giving their consent. The study protocol (IRB/ERC no.PR- 19,004) was approved by the institutional review boards of icddr,b and Stanford University.

## Results

### Neonatal hyperbilirubinemia screening

The 278 mothers who received prenatal sensitization gave birth to 273 live-born infants between November 2019 to March 2020 and October 2020 to May 2021 who were screened for neonatal hyperbilirubinemia. Five babies were not screened as three were stillborn and two died within 24 h of birth.

### Demographic information of the participants

The mean age of the parents, grandparents, and HCPs and managers who participated in the formative research were 26, 55 and 44 years, respectively. Most of the participants were female because they were available at home, were the primary caregivers, and were more interested in participating (Table 3). We invited male members from each of the families, but at the time of focus group discussions, a smaller number of males were present.

**Table 3:** Demographic Characteristics of the participants

	Formative phase			Pilot phase		
Characteristics	Grandparents (N = 29)	Parents (N = 34)	Health Practitioner (N = 9)	Grandparents (N = 16)	Parents (N = 28)	CHWs (N = 10)
Mean age, years (standard deviation)	55 (6)	26 (4.8)	44 (7.1)	58 (5.5)	33 (5.2)	29 (5.8)
Average monthly income, BDT	10,000	15,000	---	12,000	16,000	10,000
Average working experience (years)	--	--	9	--	--	2.5
**Education** Completed primary (5 years of schooling) education	8 (28%)	10 (29%)		5 (35%)	10 (35%)	
Completed secondary to higher secondary (10–12 years of schooling) education	--	22 (65%)	3 (33.3%)		14 (50%)	4 (40%)
Graduation and above	--	1 (3%)	6 (66.6%)		4 (14%)	6 (60%)
No institutional education	21(72%)	1 (3%)		11 (65%)		
**Profession** Housewife	25 (86%)	31 (91%)		9 (64%)	16 (57%)	
Agriculture/day labor in agricultural work	3 (10%)	2 (6%)		5 (36%)	4 (14%)	
Government Service			5 (70%)			
Private service			3 (30%)		8 (28%)	10 (100%)
Retired/no job	1 (3%)	1 (3%)				
**Sex** Male	2 (6%)	3 (9%)	7 (90%)	5 (35%)	12 (42%)	
Female	27 (94%)	31 (91%)	1 (10%)	9 (65%)	16 (58%)	10 (100%)

### Existing gaps in knowledge, perceptions and management of neonatal jaundice

**Fig. 3:**
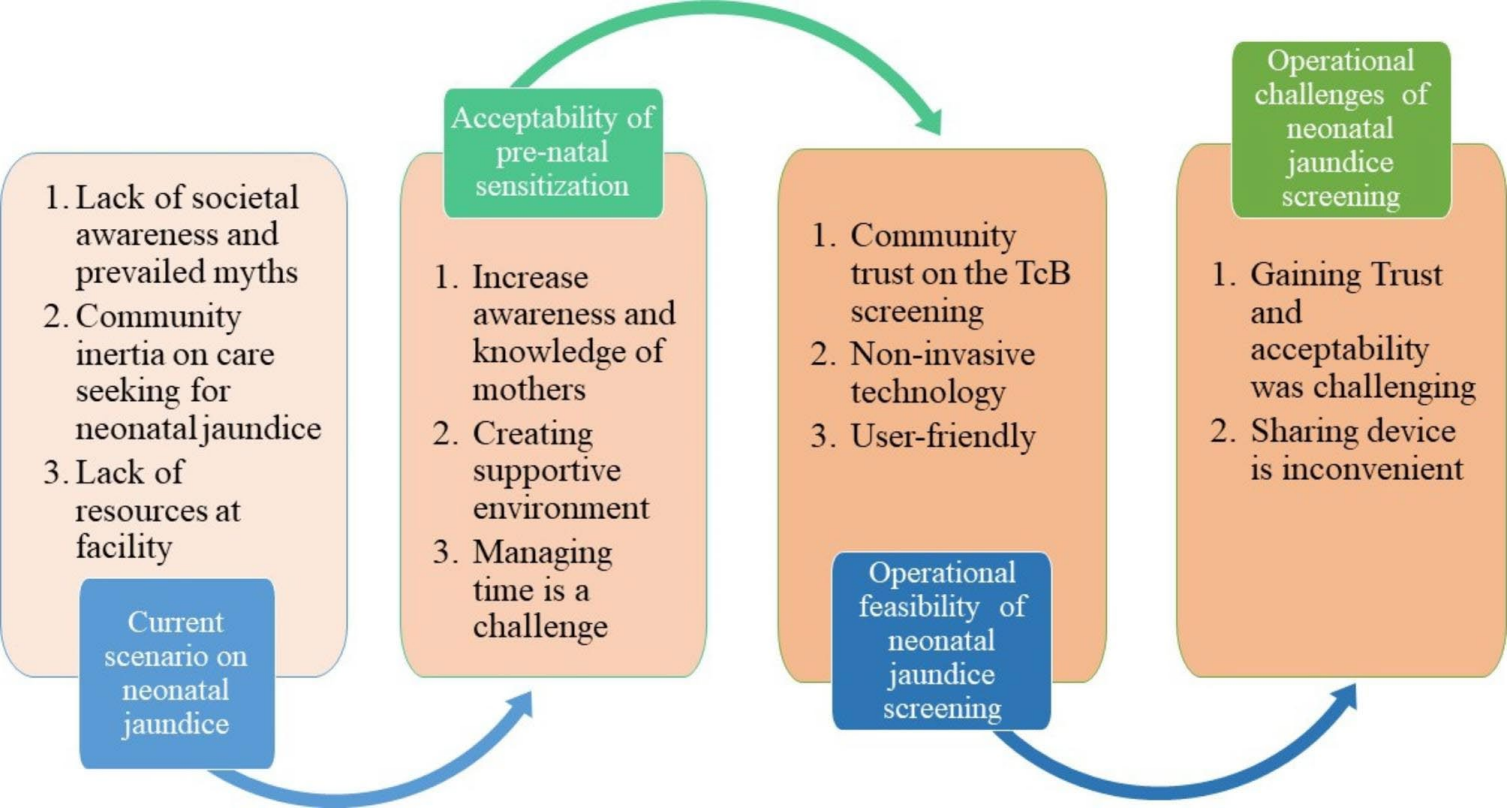
Summary of key findings of current situation, acceptability of prenatal sensitization and operational feasibility of neonatal jaundice screening

The following themes emerged under existing gaps in knowledge, perceptions and management of neonatal hyperbilirubinemia: (1) Lack of societal awareness and prevailing myths, (2) Community inertia on care-seeking for neonatal jaundice, and (3) Lack of resources for neonatal hyperbilirubinemia management (Fig. 3).

#### Lack of societal awareness and prevailing myths

The majority of the mothers and grandparents reported that they had heard about neonatal hyperbilirubinemia, but they could not explain the condition or its causes. They reported detecting jaundice through visual inspection of yellow discoloration of the skin of the newborn and yellowness of the eyes. Some of the grandparents mentioned yellowish urine as a symptom of neonatal jaundice. One grandmother during a focus group discussion said, “*If we see baby’s yellowish urine, eyes, face, we know from our experience that this is jaundice. We do not have to visit a doctor for jaundice diagnosis. We can identify those symptoms ourselves”*.

In response to the cause of neonatal hyperbilirubinemia, most of the grandparents (three-fourths) mentioned that babies get jaundice either from their mothers’ wombs or from the foods mothers eat. Some grandparents believed that eating food with turmeric or too much spice, or yellow fruit and vegetables like papayas and carrots can cause jaundice. Some mothers also reported that jaundice can spread by drinking water from the same glass of an infected person because the germs can transfer through water and infect a new person. Mothers reported that they ate white curry (without spices) as advised by family members if the baby had jaundice.

#### Community inertia on care-seeking for neonatal jaundice

Most parents and grandparents reported that they do not consider newborn jaundice as a problem requiring care from physicians. They believed it is very common after birth and is not something serious. About half of them reported that the only treatment for jaundice is “keeping baby under sunlight”. A few mothers reported that they kept the baby under sunlight but did not know that it was a treatment for jaundice. Two thirds of participants thought that jaundice could be managed at home by homeopathic and traditional medicine from informal providers. They also believed mothers’ food habits are important in treating jaundice. For example, if the mothers avoid spices and yellow fruit, babies with jaundice will recover soon. Some grandparents believed that mothers should not breastfeed their babies when the babies have jaundice.

One grandfather aged 70 years mentioned, “*We tell our daughters-in-law to intake coconut water if any of them or babies are affected by jaundice. Any adult affected by jaundice should also drink coconut water. This is common practice in our area. If the mothers have enough coconut water it will help to improve babies as well. We knew it from our parents and grandparents.”*

Most of the parents and grandparents had never heard about phototherapy or seen phototherapy devices. A couple of parents claimed to have heard the term ‘phototherapy machine’, but they did not know exactly what it was or how it works.

#### Lack of resources for neonatal hyperbilirubinemia management

HCPs and managers mentioned that most of the sub-district level hospitals lack phototherapy facilities. The sub-district hospital in the study area had a phototherapy machine, but the service was not available due to the lack of human resources. Key informants reported that HCPs used to refer infants with neonatal hyperbilirubinemia to the nearest district hospital which was located approximately 35 km away. They also expressed concerns that referring newborns presenting with jaundice and other danger signs may result in deterioration of the infant’s condition in transit. The residential medical officer reported, “*We do not manage any case of neonatal jaundice here as we don’t have a functioning phototherapy unit or trained nurses to operate this. Whenever we receive a case of neonatal hyperbilirubinemia, we refer them to Tangail district hospital, without further delay as it takes some time to go there.”*

They also reported that there is no separate ward to provide phototherapy at the sub-district hospital. Thus, if the babies are otherwise healthy, home phototherapy can be a feasible option for the treatment of neonatal jaundice. This will also eliminate the chance of contracting a hospital-acquired infection. Half of the HCPs reported that they did not receive any specialized in-service training on newborn management, such as Emergency Triage Assessment and Training (ETAT).

### Acceptability of prenatal sensitization

#### Increased awareness and knowledge of mothers on care-seeking for neonatal jaundice

The CHWs mentioned because of the intensive and theoretical training, all the mothers reported that the home-based prenatal educational sessions increased their knowledge of antenatal care, breastfeeding, newborn care and danger signs, symptoms and care-seeking for neonatal hyperbilirubinemia. They reported that knowing the symptoms and management of neonatal hyperbilirubinemia enabled them to take timely decisions for appropriate care-seeking. They learned from CHWs that during pregnancy, parents should test their blood group; if a mother has a negative blood type, the risk of neonatal hyperbilirubinemia can be increased. Mothers mentioned that the yellowish color of the palms and soles and convulsions are danger signs of neonatal hyperbilirubinemia. One mother aged 29 years (in-depth interview 11) reported, “*If the baby has jaundice, the baby gets lethargic, urine gets yellow, the stool becomes white, can’t eat and cry. The baby’s tongue, palm and becomes yellowish. Sometimes a blood test is needed”*.

One mother aged 25 years (focus group discussion 2) mentioned, “*Baby does not want to eat during illness and if they cannot suck breast milk then it is required to release breast milk. Extra time and effort should be given while breastfeeding. Expressed breastmilk can be stored in the refrigerator for some time*”.

#### Creating a supportive family and community environment

Parents mentioned that the CHWs not only provided educational sessions to mothers but also counseled other family members about the importance of healthy food for mothers and newborns, as family members often expressed concern about the nutrition of the mothers.

Grandparents mentioned that they did not know what kind of information was provided to mothers as they did not participate in the sessions with them, but the grandparents were provided health messages for the mothers’ wellbeing. Grandparents advised pregnant women to eat fish protein and leafy vegetables. They also mentioned they took the pregnant mothers to the doctors when there were any complications.

#### Managing time to attend sessions is a challenge

One-third of the mothers mentioned that sometimes it became difficult for them to attend the sessions on time. They reported that initially family members did not appreciate mothers attending the session as this prevented them from doing other chores at that time. Sometimes mothers had to do other household chores and take care of the other children, causing them to miss sessions, or they had to leave the session early for other competing responsibilities such as attending to guests. However, mothers reported that the CHWs were very supportive and often waited for the mothers or came on the next day if they missed any sessions.

### Operational feasibility of neonatal hyperbilirubinemia screening

#### The community trusted information from transcutaneous bilirubin (TcB) screening over physical examination

The majority of the CHWs reported that although they were concerned initially with how people would react to this new device, they received very positive feedback from parents and family members. CHWs explained to families that the device showed the level of jaundice after touching it over the child’s sternum, and the parents were very keen to know if the child had jaundice and its severity. CHWs also assessed for physical examination findings of elevated levels of neonatal hyperbilirubinemia, but parents did not value the physical examination findings as much as they valued the reading from the TcB device. When CHWs told parents after TcB screening that their baby had jaundice and needed phototherapy, the parents acted immediately on home or hospital phototherapy. However, when the CHWs advised the parents to go to the hospital based on identifying danger signs during a physical examination, the parents were reluctant to comply with the referral.

#### Non-invasive technology and immediate reporting were appreciated by the community

Most mothers and grandparents appreciated the bilimeter. They mentioned that the screening device is a good device as it is painless, there is no need to prick the baby and collect blood, it shows the result immediately and the baby can receive treatment without delay. The mothers also mentioned that nobody wants their baby to get hurt. As it was a painless screening method, family members also supported this. They also mentioned that taking newborns to a diagnostic center and then pricking for blood is very troublesome. In addition to that, mothers and grandparents were satisfied as the bilimeter showed the status of their baby’s jaundice immediately without any waiting time. In the diagnostic centres and hospitals, they had to wait for a couple of hours to get the results. One mother aged 33 years (in-depth interview 7) mentioned, *“My previous child had jaundice after birth, I was in a private clinic, the nurses regularly took blood to see if jaundice has improved, it was very painful, my baby cried a lot. But my second baby was tested with this new device, and it did not hurt at all.”*

#### User-friendly screening device

CHWs reported that the handheld bilimeter was user-friendly and easy to operate. They mentioned that it took only a 30-minute session to learn the operation and maintenance of the bilimeter. They did not face any technical difficulties regarding using bilimeters during the project period. The bilimeter device was very lightweight, so they could carry it easily during home visits. One CHW aged 26 years (in-depth interview 14) mentioned, “*The bilimeter was easy to use and maintain. We were provided with training on how to use this. I used to clean the head of the device with an alcohol pad every time before using it, I then turned on the power, placed it vertically over the middle of the sternum and pressed it until I heard a click sound*.”

### Operational challenges faced

#### Gaining trust and acceptability from the community was challenging

Most CHWs mentioned that initially the community members were not very supportive of home-based screening of neonatal hyperbilirubinemia by CHWs with a portable bilimeter because they were not confident about CHWs’ competence. The mothers did not want to participate in the sessions as they thought them a waste of time. However, these negative attitudes changed over time when families realized the ease of using the bilimeter to objectively screen and identify newborns with neonatal hyperbilirubinemia. The study team organized community meetings to introduce the program activities to community members and leaders. CHWs reported that those community engagement activities were helpful to gain community support for neonatal hyperbilirubinemia screening at home.

#### Sharing the device was inconvenient

Sharing of one device by three CHWs was a challenge for real-time screening of newborns at the household level. The devices were kept at the field office, and the CHWs used to carry the device from the office. They also reported that sometimes more than one CHW needed to use a single bilimeter on the same day. They accompanied the other CHWs during their visits and then collected the bilimeter after completion of their assessments. The CHWs reported that it would be more convenient if they had one device per CHW so that they could keep it with them always.

## Discussion

Household CHW-led neonatal hyperbilirubinemia screening with a transcutaneous bilimeter was acceptable to families in a rural Bangladesh community and resulted in successful referrals for hospital- or home-based care. CHWs, parents and grandparents expressed enthusiasm for neonatal hyperbilirubinemia screening with a TcB device because of its non-invasive technology, easy and pain-free use, and instant and objective results. CHWs gained trust of families using the device; families could see that device enabled CHWs to make an objective measurement that required minimal skill and could be done at home instead of in a health facility.

Objective measurement with the digital transcutaneous bilimeter helped build trust when CHWs recommended facility-level referral and evaluation in comparison to identification of clinical signs by physical examination. This is an important feature as CHW referrals for facility-level care based on physical assessment for newborn danger signs in a previous study in Bangladesh resulted in only one third of families successfully completing the referral. [26] Other bilirubin screening devices such as the icterometer that require physical exam assessment and interpretation by the user may also result in low referral completion rates if deployed by a CHW because the parent may not trust the CHW’s physical exam assessment and ignore the referral recommendation. [27] Parents also have shown reluctance to comply with invasive blood sampling to test for neonatal hyperbilirubinemia due to newborn pain, inconvenience of collection in a laboratory and waiting for the results. [28–31] Unsuccessful referral leading to a lack of treatment would limit the health impact of a neonatal hyperbilirubinemia screening intervention.

Assessments of acceptance and operational feasibility revealed that CHWs were effective in increasing appropriate care-seeking behaviors for newborns by educating mothers on the importance of appropriate care-seeking. Through prenatal sensitization, caregivers and family members were sensitized about the importance of early detection of neonatal hyperbilirubinemia through screening, which reflects in high coverage of neonatal hyperbilirubinemia screening. Previous studies in South Asian countries also showed home-based assessments and screening can increase coverage of maternal and newborn health care-seeking. [32–34]

The formative study revealed there were many misconceptions about neonatal hyperbilirubinemia including the causes, the potential need for facility-level care and the potential health risks for newborns. Parents and grandparents could not explain what neonatal hyperbilirubimemia was, beyond the yellowish color of skin and eyes. Participants did not have adequate knowledge about the cause of jaundice, nor the health risks for newborns. It was thought that babies become jaundiced from food eaten by mothers and that if the mothers consumed coconut juice and avoided spices, they could reduce the jaundice levels in their newborns. These findings were reflected in other studies where mothers believed consumption of peanuts and palm oil by mothers can cause jaundice in newborns. [35, 36] Other studies conducted in rural communities of India, Nigeria and Ghana showed similar findings [16, 36–41] and indicate that neonatal jaundice-related knowledge and misconceptions prevail in different cultural contexts. The majority of parents and grandparents did not believe that neonatal hyperbilirubinemia is a disease requiring care from HCPs. Putting babies under sunlight is the most practiced treatment method by mothers and grandparents. In addition, ayurvedic and homeopathy treatment were also preferred treatments for neonatal jaundice. Caregiver misconceptions regarding health risks due to neonatal hyperbilirubinemia are consistent with low neonatal hyperbilirubinemia care-seeking in other low-resource settings. [16, 35, 39]

Prenatal sensitization has been shown to be a feasible way to improve caregivers’ knowledge of antenatal care, vaccination, breastfeeding practice, and HIV awareness. [34, 42, 43] In our study, CHWs faced some initial challenges while conducting home-based education, primarily due to a lack of support from family members to spare mothers for the sessions. Community engagement activities eased this challenge and helped to ensure support from the family and community. Previous community-based interventions have shown that community engagement activities increase the coverage and uptake and reduce the barriers in intervention implementation. [44]

Our study revealed that CHWs were confident in delivering the messages because they had adequate theoretical and hands-on training. CHWs were satisfied with their job beyond monetary compensation because they were honored and considered important people in the community. Community appreciation and encouragement played an important role in motivating CHWs to deliver their service successfully, as has been reported elsewhere.[44] Community-based cardiovascular and HIV screening programs and maternal health care service delivery programs have shown that community trust and behavioral incentives motivated CHWs for delivery of their job along with financial incentives. [45–49]

There were some limitations of this study. Our results should be interpreted cautiously because CHWs were assigned to a limited number of households. Further studies should focus on estimation of workload and cost effectiveness of such screening programs. The capital cost of bilimeters that we used in our study was high (USD 3500); however, after the initial purchase, ongoing cost to charge the device is minimal. Further studies on the cost implications of this screening program and alternative approaches are needed. The cost of transcutaneous bilimeters may decrease overtime as well.

## Conclusions

A household-based, CHW-led neonatal hyperbilirubinemia screening program was highly acceptable for parents, grandparents, HCPs and CHWs in a population with a relatively high percentage of home births and low coverage of timely postnatal care. Neonatal hyperbilirubinemia was often not recognized as a medically important condition by parents and grandmparents. In a low-resource area, where the availability and cost of serum bilirubin measurement was a major obstacle to screening newborns for neonatal hyperbilirubinemia, CHW-led household hyperbilirubineamia screening with the handheld TcB provided an acceptable and non-invasive alternative and enabled access to newborns born at home who do not typically receive postnatal care. CHWs were enabled to rapidly identify newborns with neonatal hyperbilirubinemia and facilitate treatment in the hospital or at home. CHW-led prenatal sensitization can increase awareness of mothers and family members to create a supportive environment to improve coverage of neonatal hyperbilirubinemia screening. Further work should explore how a CHW-led neonatal hyperbilirubinemia screening program could be integrated into care delivery systems in LMICs.

## Data Availability

Institutional Review Board approval was obtained for public sharing and presentation of data on a group level only. To maintain participants’ anonymity and confidentiality, the data set generated during the study will not be publicly available but is available from the corresponding author on reasonable request.

## Abbreviations

LMIC: Low- and Middle-Income Countries
CHW: Community Health Worker
UHC: Upazilla Health Complex (Sub-district hospital)
TcB: Transcutaneous Bilirubin
TsB: Total Serum Bilirubin
HCP: Health Care Providers
